# Supplementary material for: Do Food and Meal Organization Systems in Polish Primary Schools Reflect Students’ Preferences and Healthy and Sustainable Dietary Guidelines? The Results of Qualitative Research for the Junior-Edu-Żywienie (JEŻ) Project
Source: Foods. 2023 Dec 22;13(1):61. doi: 10.3390/foods13010061 (PMC10778281; doi:10.3390/foods13010061)
Supplement: Supplementary file 1 [file foods-13-00061-s001.zip › foods-2771724-supplementary.pdf]

INDIVIDUAL IN-DEPTH INTERVIEW (IDI) SCRIPT

Participants of the interview: 24 of Headmasters, 24 representatives of people employed in school canteens or catering companies organizing meals in schools
IDI duration: 60 minutes

1. Introduction to the interview (5 minutes)

- Interviewer's introduction
- Presentation of the purpose of conducting the interview
- Explanation that the interview will be recorded, and the recorded content is confidential and will be used for research purposes
- Asking participants if they have any questions - interviewer's responses and clarification of any doubts
- Informing about the duration of the meeting: up to 1 hour.

2. The current state of nutrition organization at school (15-20 minutes)

- How is children's nutrition organized at school? (school canteen, catering, other) Who is responsible for organizing and planning nutrition at school?
- Is there a food tuck shop at school? Are there vending machines with food products (sweets, drinks, other)?
- What percentage of children eat meals at school? Is this distributed differently among students at different grade levels; e.g., students of grades 1-3 use it more often, and older students use it less often? Why don't other students benefit from school meals?
- Are students satisfied with school nutrition? What dishes do children like? What dishes are disliked? What food do they most often leave on their plates? Do children like to eat vegetables?
- Are teachers and students' parents satisfied with the school nutrition?
- What dietary recommendations are taken into account when nutrition planning?
- Is it monitored by school which children do not eat meals? Are there programs at school to support children from the poorest families?
- What is the role of the parents' council in organizing school nutrition?
- Do school employees eat meals organized at school? Are they satisfied?
- What is the scale of food waste at school? What is thrown away most often? Have any actions been implemented to reduce the scale of food waste? What are these activities?
- How is waste collection organized?
- How do you imagine the ideal school nutrition system?

Questions regarding the operation of canteens.

- How many people are employed in the nutrition organization?
- What does work organization look like? What are the positions? What is the working mode? (working hours, possible shifts, number of positions)
- Where are the food products delivered from? How many suppliers are there?
- What did the canteen supplies look like during the COVID-19 pandemic?
- What problems have occurred during the COVID-19 pandemic?

„The task entitled 'Conducting scientific research in the field of nutrition of children and adolescents, developing and implementing a nutritional education program for pupils of grades 1-6 of primary schools. Acronym: Junior-Edu-Żywienie (JEŻ)'. The study was financially supported by the Polish Ministry of Education and Science (MEiN/2022/DPI/96 of 7 March 2022).

- What additional actions has the school taken during the pandemic?
- In the case of 1-3 grades, is someone from the teaching staff present when students are eating meals?

Questions about catering

- Since when does school nutrition is provided by a catering company?
- How long does the cooperation with the catering company currently delivering meals to the school last?
- How does cooperation look like? Is the school satisfied with the cooperation with this company?
- Who plans the menu? What is the role of school?
- What time are meals delivered? What time are meals served to children? Are school meals heated? How are meals served to children? Are the people serving meals school employees?
- What would the school change in the catering organization (e.g., catering company, other)?

Providing nutrition during the COVID-19 pandemic

- How has the COVID-19 pandemic affected the functioning of catering? How did the quarantine of individual grades affect the organization of catering? How and when was information about the need to quarantine or isolate individual classes communicated?
- What actions have been taken at school to ensure students' nutrition during the COVID-19 pandemic?

---

3. Assessment of meals and the current state of nutrition organization at school (10 minutes)

- Is information about satisfaction with meals obtained among children, parents and school employees, and how?
- How can meals served at school be assessed in terms of:
  - quality,
  - nutritional value,
  - compliance with nutritional recommendations?
- Are there consultations with a dietitian regarding meal planning at school? Does the person creating the menu have sufficient knowledge/education on this topic? Is it possible to meet the nutritional recommendations at the school's proposed dietary rate?
- Where do people responsible for nutrition at school get their knowledge about nutritional recommendations? Do they have appropriate education and training in this area? Do they supplement their knowledge about current recommendations on the nutrition of children and adolescents, e.g., by participating in training; courses?

---

4. Identification of problems in the organization of school nutrition? (10 minutes)

- Are there any problems related to the organization of nutrition at school? (supplies, staffing problems, problems with catering facilities, kitchen and dining room equipment, financial problems)
- Does the school benefit from funding from local authorities? (e.g., parents pay only for the so-called "boiler insert", and the rest is paid by the commune or city)

„The task entitled 'Conducting scientific research in the field of nutrition of children and adolescents, developing and implementing a nutritional education program for pupils of grades 1-6 of primary schools. Acronym: Junior-Edu-Żywienie (JEŻ)'. The study was financially supported by the Polish Ministry of Education and Science (MEiN/2022/DPI/96 of 7 March 2022).

5. Prospects for organizing school nutrition? (10 minutes)

- Are there any planned changes in the organization of nutrition at school?
- Does the school need support in organizing meals? From the local authorities? Ministries? or other institutions?

6. Summary and thanks for participating in the interview (5 minutes)

- A brief summary of the interview
- Is there anything else anyone would like to add?
- Thank you for participating in the interview

„The task entitled 'Conducting scientific research in the field of nutrition of children and adolescents, developing and implementing a nutritional education program for pupils of grades 1-6 of primary schools. Acronym: Junior-Edu-Żywienie (JEŻ)'. The study was financially supported by the Polish Ministry of Education and Science (MEiN/2022/DPI/96 of 7 March 2022).
